# Semantic Relatedness Emerges in Deep Convolutional Neural Networks Designed for Object Recognition

**DOI:** 10.3389/fncom.2021.625804

**Published:** 2021-02-22

**Authors:** Taicheng Huang, Zonglei Zhen, Jia Liu

**Affiliations:** ^1^State Key Laboratory of Cognitive Neuroscience and Learning and IDG/McGovern Institute for Brain Research, Beijing Normal University, Beijing, China; ^2^Beijing Key Laboratory of Applied Experimental Psychology, Faculty of Psychology, Beijing Normal University, Beijing, China; ^3^Department of Psychology, Tsinghua University, Beijing, China

**Keywords:** deep convolutional neural network, semantic relatedness, WordNet, perceptual experience, conceptual guidance

## Abstract

Human not only can effortlessly recognize objects, but also characterize object categories into semantic concepts with a nested hierarchical structure. One dominant view is that top-down conceptual guidance is necessary to form such hierarchy. Here we challenged this idea by examining whether deep convolutional neural networks (DCNNs) could learn relations among objects purely based on bottom-up perceptual experience of objects through training for object categorization. Specifically, we explored representational similarity among objects in a typical DCNN (e.g., AlexNet), and found that representations of object categories were organized in a hierarchical fashion, suggesting that the relatedness among objects emerged automatically when learning to recognize them. Critically, the emerged relatedness of objects in the DCNN was highly similar to the WordNet in human, implying that top-down conceptual guidance may not be a prerequisite for human learning the relatedness among objects. In addition, the developmental trajectory of the relatedness among objects during training revealed that the hierarchical structure was constructed in a coarse-to-fine fashion, and evolved into maturity before the establishment of object recognition ability. Finally, the fineness of the relatedness was greatly shaped by the demand of tasks that the DCNN performed, as the higher superordinate level of object classification was, the coarser the hierarchical structure of the relatedness emerged. Taken together, our study provides the first empirical evidence that semantic relatedness of objects emerged as a by-product of object recognition in DCNNs, implying that human may acquire semantic knowledge on objects without explicit top-down conceptual guidance.

## Significance

The origin of semantic concepts is in a long-standing debate, where top-down conceptual guidance is thought necessary to form the hierarchy structure of objects. However, an alternative hypothesis argues that semantic concepts derive from the perception of natural environments. Here, we addressed these hypotheses by examining whether deep convolutional neural networks (DCNNs), which only have abundant perceptual experience of objects, can emerge the semantic relatedness of objects with no conceptual relation information was provided. We found that in the DCNNs representations of objects were organized in a hierarchical fashion, which was highly similar to WordNet in human. This finding suggests that top-down conceptual guidance may not be a prerequisite for human learning the relatedness among objects; rather, semantic relatedness of objects may emerge from the perception of visual experiences for object recognition.

## Introduction

Objects in this world are complicated. Variations of objects (e.g., orientation, size, shape and color) create challenges for human to flexibly recognize and categorize them (Logothetis and Sheinberg, 1996). To survive in such difficult and diverse environments, humans learn to characterize objects into a rich and nested hierarchical structure, which finally evolves into semantic concepts (Tanaka, 1996; Yamins et al., 2014). However, how the hierarchically-structured semantic concepts are formed is still hotly debated.

Two hypotheses have been proposed. One hypothesis (Mahon and Caramazza, 2009; Leshinskaya and Caramazza, 2016) suggests that semantic concepts are only formed and accessed through abstract symbols that are independent of perceptual experiences. Supporting evidence comes from studies on congenitally blind people, whose core semantic retrieval system in the frontal-temporal cortex can still be activated for retrieving visually-experienced semantic information (Noppeney et al., 2003; Noppeney, 2007). In addition, functional brain imaging studies find that supramodal regions in the ventral temporal occipital cortex (e.g., superior occipital, inferior and superior parietal areas) are also involved in processing objects in blind individuals (Lambert et al., 2004; Ricciardi et al., 2014). Therefore, perceptual experiences seemed not necessary for the emergence of semantic concepts.

An alternative hypothesis argues that the development of semantic concepts derives from perception of natural environments (Sloutsky, 2003; Roy, 2005; Barsalou, 2008). For example, in a word/no word match-to-sample task, Imai et al. (1994) decouple taxonomic and perceptual similarity of words, and find that younger children rely on the visual property of objects, rather than taxonomic concepts, in response to novel words. More direct evidence comes from a study on 10-month-old infants who learn new words by the perceptual salience of an object rather than social cues provided by the caregivers (Pruden et al., 2006). That is, perceptual features are needed to form semantic concepts.

One inevitable limitation of these studies is that perceptual experiences and conceptual guidance are tightly intermingled during the development; therefore, it is impossible to examine one factor with the other controlled. In contrast, the advance of deep convolutional neural networks (DCNNs) provides a perfect model to examine how semantic relatedness is formed (Khaligh-Razavi and Kriegeskorte, 2014; Jozwik et al., 2017; Peterson et al., 2018). On one hand, DCNNs have abundant visual experiences on objects, as with the presence of millions of natural images, the DCNNs learn to extract critical visual features to classify objects into categories as perfectly as human. On the other hand, during the training, the relation among object categories is not provided in the training task or in the supervised feedback. Therefore, conceptual guidance is completely absent in the DCNNs. With such characteristics of the DCNNs, here we asked whether semantic relatedness among object categories was able to emerge with no top-down conceptual guidance.

To address this question, we used a typical DCNN, AlexNet, which is designed for classifying objects into 1,000 categories in ImageNet. Specifically, we first measured whether the representations of some object categories were more similar than their relation to others, which formed a hierarchical structure of object categories as a whole. We reasoned that if a stable and well-organized hierarchical structure was observed, the hypothesis of the necessity of conceptual guidance in forming the semantic relatedness was challenged.

## Materials and Methods

### The ImageNet Dataset

We used the ILSVRC2012 dataset (Russakovsky et al., 2015) as the image stimulus (http://image-net.org/challenges/LSVRC/2012/). Both training and validation datasets were used in this study. The ILSVRC2012 training dataset contains about 1.2 million images with labels from 1,000 categories. The validation dataset contains 50,000 unduplicated images that belong to the same 1,000 categories as the training dataset.

**Figure 1:**
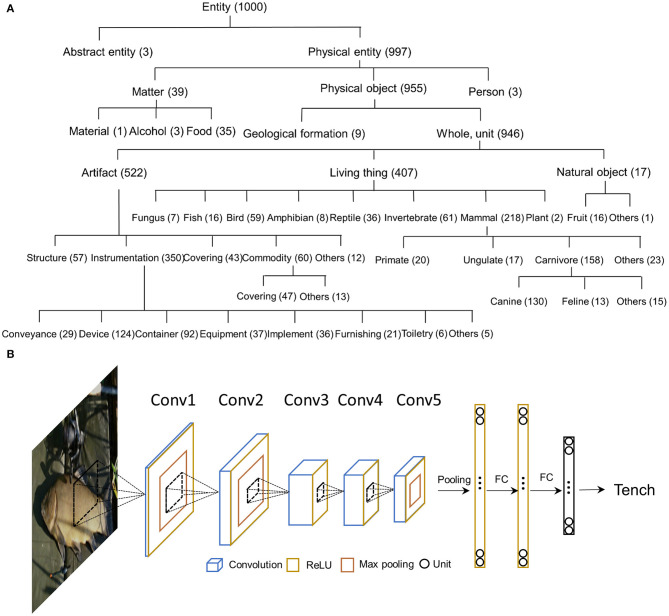
**(A)** The hierarchical structure of 1,000 object categories in the WordNet. All categories were derived from an ontology root (e.g., entity), and most of them are the subsets of the physical entity. The 1,000 categories cover a wide range of physical objects, making it suitable to study the emerge of object relatedness. Numerals after each word are the number of categories belonging to this superordinate category. **(B)** The architecture of AlexNet. The AlexNet includes 8 layers of computational units stacked into a hierarchical architecture: the first 5 are convolutional layers, and the last 3 layers are fully connected for category classification.

Each object category from ILSVRC2012 dataset corresponds to one semantic concept in the WordNet (Deng et al., 2009). Semantic concepts are described with multiple words or phrases, coined as “synonym sets” or “synset” in abbreviation. The synsets used in the ILSVRC2012 are selected from WordNet, and none has a parent-child relation with others. All 1,000 synsets have the same ontology root (i.e., entity) and most of them are subsets of the superordinate synset of physical entity. Specifically, 3 synsets belong to abstract entity (e.g., bubble, street sign, and traffic light), 39 synsets belong to matter (e.g., menu), 9 synsets belong to geological formation (e.g., cliff), 517 synsets belong to artifact (e.g., abacus), 407 synsets belong to living things (e.g., tench), and 16 synsets belong to fruits (e.g., strawberry). As shown in Figure 1A, the 1,000 synsets are organized in a hierarchical structure based on the WordNet.

### Deep Convolutional Neural Networks (DCNNs)

Six fully-pretrained DCNNs from three DCNN families were used to examine whether the emergence of semantic relatedness was a general feature of DCNNs. All DCNNs were pretrained on ImageNet with 1.2 million images for the classification of 1,000 object categories. The models were downloaded from PyTorch model Zoo (https://pytorch.org/docs/stable/torchvision/models.html).

#### AlexNet

AlexNet consists of 8 layers of computational units stacked into a hierarchical architecture, with the first 5 convolutional layers and the last 3 fully-connected layers for category classification. Rectification (ReLU) non-linearity is applied after all layers except for the last fully-connected layer (Figure 1B).

#### VGG

Two VGG networks, including VGG11 and VGG19, were used to examine whether the number of layers was critical for the emergence of semantic relatedness. VGG11 and VGG19 include 11 and 19 weight layers, respectively, with the first 8 and 16 convolutional layers and the last 3 fully-connected layers. All hidden layers are equipped with the ReLU non-linearity.

#### ResNet

Three ResNet, including ResNet18, ResNet50, and ResNet101 were used to examine the effect of residue blocks on the emergence of semantic relatedness. ResNet18, ResNet50, and ResNet101 include 18, 50, and 101 weight layers, respectively, with all convolutional layers except for the last fully-connected layer. For every two convolutional layers, a residue block is constructed by inserting a shortcut connection. ReLU nonlinearity is applied within these residue blocks.

### The Semantic Similarity of Category in WordNet

The semantic similarity of the 1,000 object categories was evaluated by the WordNet 3.0 (Miller, 1995), which is one of the most popularly-used and largest lexical databases of English. In WordNet, the lexical hierarchy is connected by several superordinate synsets in semantic relations, providing a hierarchical tree-like structure for the 1,000 synsets.

We measured the semantic similarity between each pair of the 1,000 synsets using Wu and Palmer's similarity (Wu and Palmer, 1994), which computed the similarities between concepts in an ontology restricted to taxonomic links. This measure is given by:

$$\begin{array}{l} {\text{Sim}}_{WP}(\text{X},\text{Y})=\frac{2N}{N_{1}+N_{2}} \end{array}$$

Where N_1_ and N_2_ are the depth between the concepts X, Y and the ontology root (i.e., “entity” in WordNet) and N is the depth between the least common subsume (i.e., most specific ancestor node) and the ontology root.

### Representation Similarity of Categories in DCNNs

Responses to each image were extracted from all of the convolutional layers and the last fully-connected layer using the ILSVRC2012 validation dataset with the DNNBrain toolbox (Chen et al., 2020) (https://github.com/BNUCNL/dnnbrain). No ReLU was performed for the responses. Responses of stimulus from the same category were averaged to make a response pattern for this category. The category similarity of a layer was measured as correlations of response patterns between each of two categories. In addition, correspondence between the category representational similarity from the DCNNs and the WordNet semantic similarity was calculated to measure the extent to which the relatedness of objects in the DCNNs was similar to that in humans.

### The Development of the Relatedness in DCNNs

To investigate how the hierarchical structure of objects emerged in the AlexNet, we retrained it from scratch with about 1.2 million images that belong to the 1,000 categories from the ImageNet training dataset (Deng et al., 2009) using the PyTorch toolbox (Paszke et al., 2019). The network was trained for 50 epochs, with the initial learning rate as 0.01 and a step multiple of 0.1 every 15 epochs. The parameters of each model were optimized using stochastic gradient descent with the momentum and weight decay was fixed at 0.9 and 0.0005, respectively. Each input image was transformed by random crop, horizontal flip, and normalization to improve the training effect of the network. During the training progression, object classification accuracy was evaluated in predicting the category of 50,000 images from the ILSVRC2012 validation dataset in each epoch. In the end, the top-1 and top-5 accuracies for the AlexNet were 51.0% and 74.5%.

During the training progression, we input images from the ILSVRC2012 validation dataset by simply feedforwarding in each epoch to get the activation responses, and then averaged responses within each category and computed the similarity between each pair of categories for the category similarity. Correspondence between the category similarity from the AlexNet and the WordNet semantic similarity in each training stage was measured to evaluate how similar the relatedness of objects was between the AlexNet and human.

To reveal at which semantic level the category similarity from the AlexNet showed better correspondence to the WordNet semantic similarity, the category similarity from the AlexNet was measured at a coarse level and a fine-grained level, respectively. In particular, we first manually selected 19 superordinate concepts (i.e., food, fungus, fish, bird, amphibian, reptile, mammal, invertebrate, conveyance, device, container, equipment, implement, furnishing, toiletry, covering, commodity, structure, and geological formation) that covered most of the 1,000 categories by referring to the WordNet hierarchical relationship, then grouped categories into these superordinate concepts. The coarse-grained correspondence was measured as the correlation between the AlexNet category similarity and the WordNet semantic similarity in 19 superordinate concepts. In turn, the similarity among superordinate concepts was calculated by averaging the category representation similarities from each pair of superordinate concepts. The fine-grained correspondence was measured as the averaged correspondence between the AlexNet category similarity and the WordNet semantic similarity within each superordinate concept.

### Effect of Object Co-occurrence to the Formation of Semantic Relatedness

We examined the effect of object co-occurrence in images on the emergence of semantic relatedness. To do this, annotations of object bounding boxes were collected from http://image-net.org/download-bboxes, which were annotated and verified through Amazon Mechanical Turk. To match results from the previous section, bounding boxes of the same 1,000 categories from the ILSVRC2012 dataset were selected, including 544,546 images and corresponding bounding boxes from the ILSVRC2012 training dataset, plus 50,000 images and corresponding bounding boxes from the ILSVRC2012 validation dataset.

Object bounding boxes provide information to distinguish objects from the background in each image. Pixels outside the object bounding boxes in each image were labeled as background, which was removed by setting to 255 (i.e., white color). In addition, for images containing multiple object bounding boxes (i.e., multiple objects), we randomly selected one of the object bounding boxes from these images, and retained the object within the box. Taken together, only one single object of an image remained, excluding the possibility of object co-occurrence as a source for the emergence of semantic relatedness.

We retrained an AlexNet with these single-object images using the Pytorch toolbox for 50 epochs. The top-1 and top-5 accuracies for the single-object AlexNet were 46.7% and 72.0%. Lower prediction accuracy was likely due to fewer images were used for training. Representational similarity of categories in the single-object AlexNet was measured with responses from the last fully-connected layer, and then compared with representation similarity of categories in the pre-trained AlexNet. The developmental trajectory of the single-object AlexNet was also evaluated in each training stage.

### Effect of Task Demands on Semantic Relatedness

The effect of task demands on semantic relatedness was examined by re-training AlexNet to classify objects at superordinate levels (e.g., the living thing vs. artifact) as compared to the original AlexNet mainly at the basic level (e.g., traffic light, crane).

One superordinate classification occurred at the highest level of the WordNet: the living thing and the artifact, which consisted of 958 object categories from the ILSVRC2012 dataset. The other superordinate classification occurred at an intermediate level, which consisted of 19 superordinate categories (fungus, fish, bird, amphibian, reptile, canine, primate, feline, ungulate, invertebrate, conveyance, device, container, equipment, implement, furnishing, covering, commodity, and structure). They together consisted of 866 object categories, which were the subset of the 958 categories contained in the superordinate categories of living thing and the artifact. To match the number of object categories, here we used 866 object categories in both superordinate classification tasks, which included 1,108,643 images from the ILSVRC2012 training dataset and 43,301 images from the ILSVRC2012 validation dataset.

The AlexNet for superordinate classification shared the identical architecture as the original AlexNet, except that one extra FC layer was appended to the FC3 layer (i.e., the last FC layer of the original AlexNet). The extra FC layer was designated for different superordinate classification tasks, as the AlexNet for two superordinate categories (AlexNet-Cate2) had two output units, and the AlexNet for 19 intermediate categories (AlexNet-Cate19) had 19 output units. Besides, since a new FC layer was appended to the original AlexNet that may change the dynamics of the network, we also built an AlexNet with an extra FC layer that included 1,000 output units (AlexNet-Cate1000) as the original one. The AlexNet-Cate1000 was designated for validation and for comparison with the AlexNet-Cate2 and AlexNet-Cate19.

The new AlexNets (i.e., AlexNet-Cate2/Cate19/Cate1000) were trained using the Pytorch toolbox for 50 epochs. The top-1 accuracy (top-5 accuracy) were 94.7% (100.0%), 68.7% (95.6%) and 49.0% (73.6%), respectively. Representational similarities of categories in the new AlexNets were measured with responses from all of their layers. Category similarity from the AlexNet-Cate1000 was compared with that of the original AlexNet to validate if they shared a similar hierarchy of semantic relatedness.

### Data Availability

All data and code underlying our study and necessary to reproduce our results are available on Github: https://github.com/helloTC/SemanticRelation.

## Results

**Figure 2:**
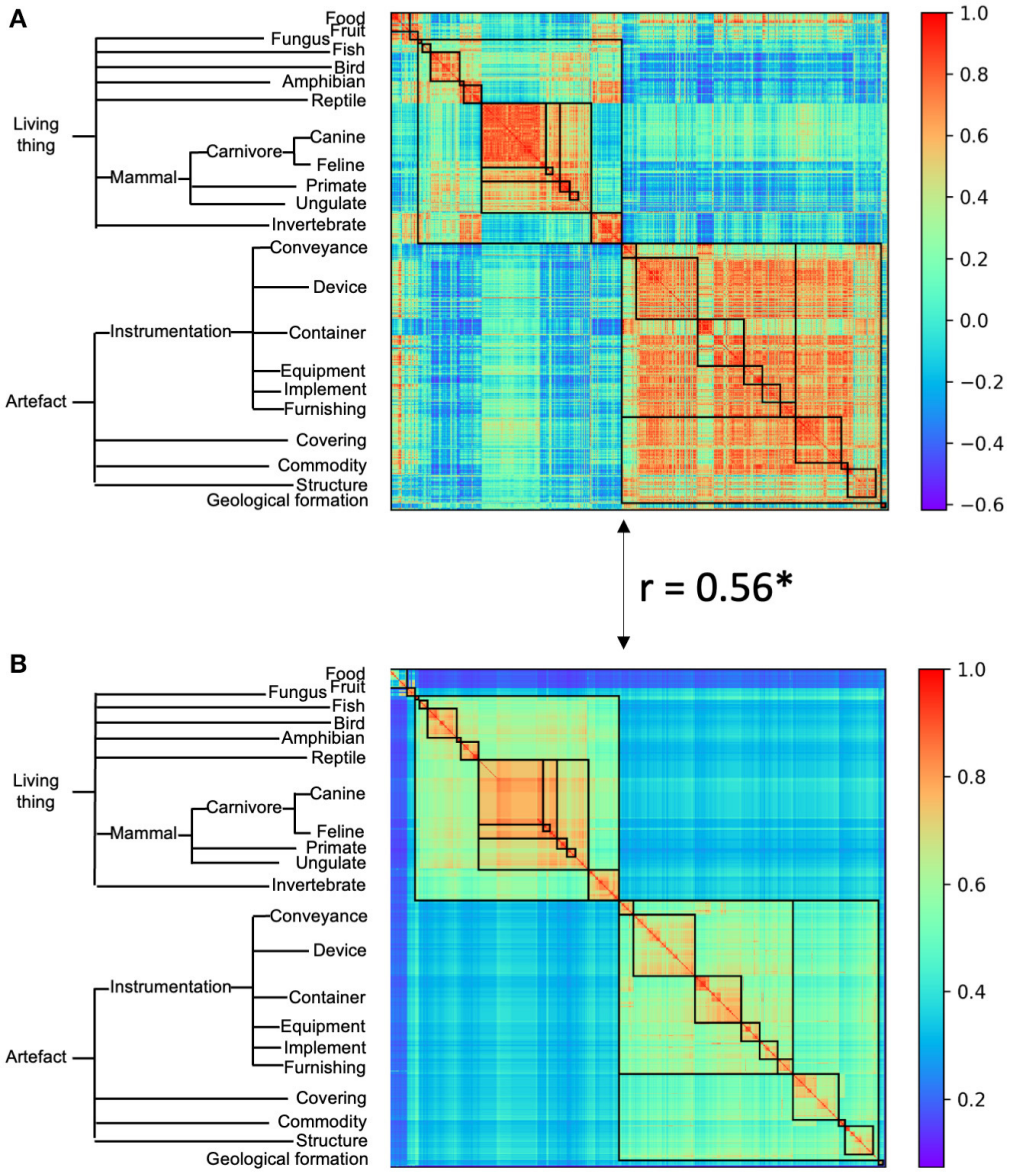
The category representational similarity of the AlexNet **(A)** and the semantic similarity of WordNet hierarchy **(B)**. Categories were ordered according to the WordNet semantic hierarchy. A simplified hierarchical structure was shown as an indicator of superordinate categories in WordNet semantic similarity. For the ease of comparison between AlexNet's category similarity and WordNet semantic similarity, categories belong to the same superordinate category were marked with a black box. The AlexNet category similarity showed good correspondence to the WordNet semantic similarity. Asterisk denotes *p* < 0.001.

We first evaluated whether there was a hierarchical structure among object categories in the AlexNet, which was trained to classify object categories from the ImageNet containing no relation information among objects. For this, responses from the last fully-connected layer of the AlexNet (i.e., FC3) were averaged across images of each category as the response pattern for this category, and the similarity between two categories was calculated as the correlation between their response patterns. A great variance in similarity was observed, with the highest similarity between object toy poodle and object miniature poodle (*r* = 0.99), the lowest between object snail and object fur coat (*r* = −0.62), and the mean similarity of *r* = 0.21. The variance in similarity observed was significantly larger than variance from a randomized structure (permutation analysis, *p* < 0.001), suggesting that objects were structurally organized (Figure 2A, left). A close inspection of Figure 2A revealed two large clusters, one is living things and the other artifacts. Within each cluster, there are sub-clusters, as within-cluster variance was smaller than that of neighboring sub-clusters. The nested structure in similarity suggests that a hierarchical relation among objects emerged in the AlexNet without conceptual guidance.

**Figure 3:**
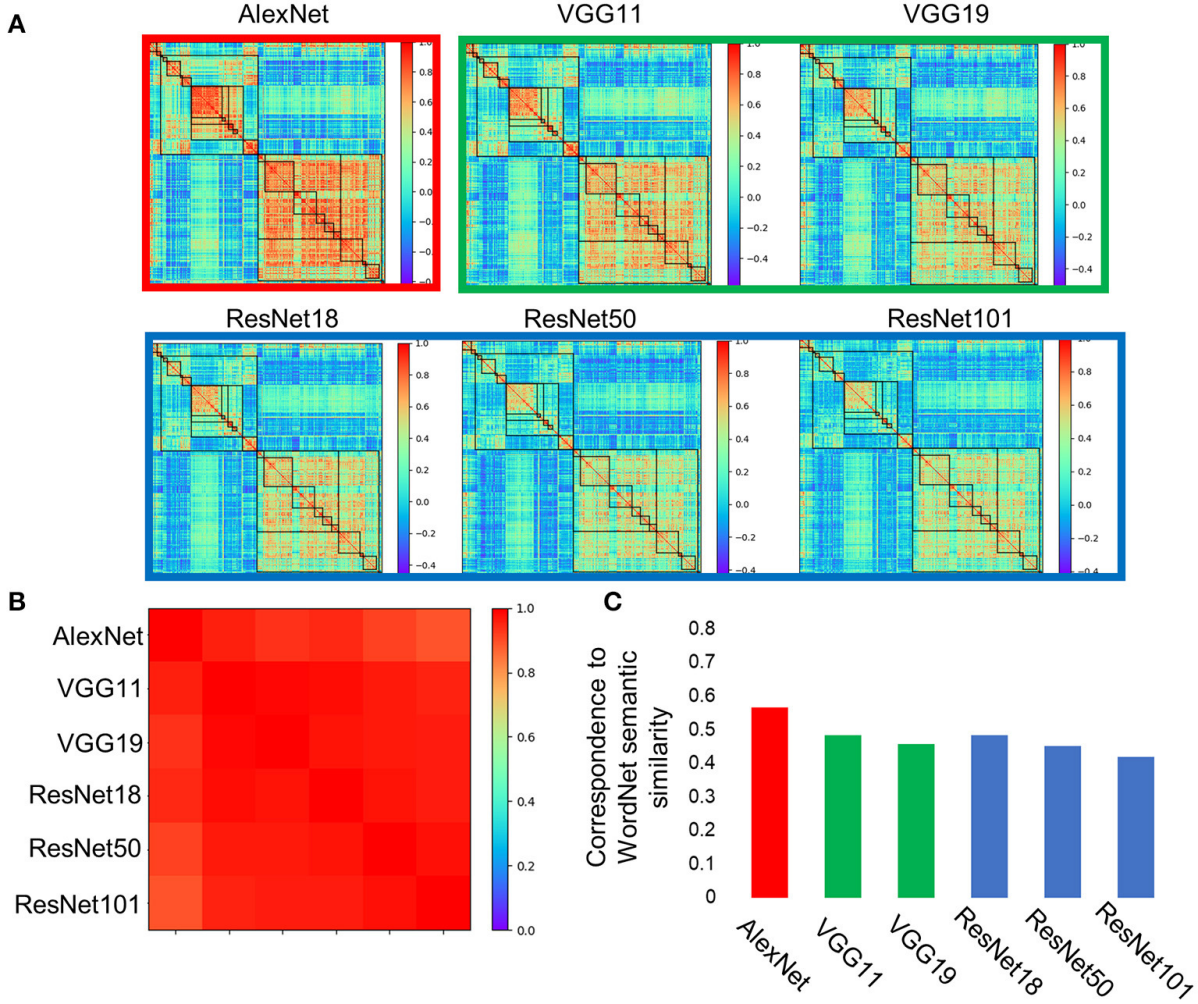
Category representations in the DCNNs were stabled across architectures. **(A)** Categorical representations from AlexNet, two VGGs (i.e., VGG11 and VGG19), and three ResNets (i.e., ResNet18, ResNet50, and ResNet101) showed consistent hierarchical relation of object categories. **(B)** The hierarchical relations that emerged in these DCNNs were almost identical among each other. **(C)** Correspondences between the hierarchical relation among objects in the DCNNs and semantic similarity of WordNet in humans were significant.

Similar nested structures of objects were also observed in DCNNs with different architectures (e.g., layer number and kernel size), including two VGGs and three ResNets, which are designed for the same task (Figure 3A). Importantly, the hierarchical relations of the object categories that emerged from the VGG family and ResNet family were almost identical to that from the AlexNet (*r* > 0.89 for all DCNNs tested, Figure 3B), implying that the emerged hierarchical relation among object categories was invariant to implementations, but rather resulted from inherent properties of the stimulus and the task that DCNNs received and performed. Because human brains used images from the same physical world to perform the same task, one intuitive thought is that the hierarchical relation observed in the DCNNs may be similar to the semantic relatedness of objects in human.

**Figure 4:**
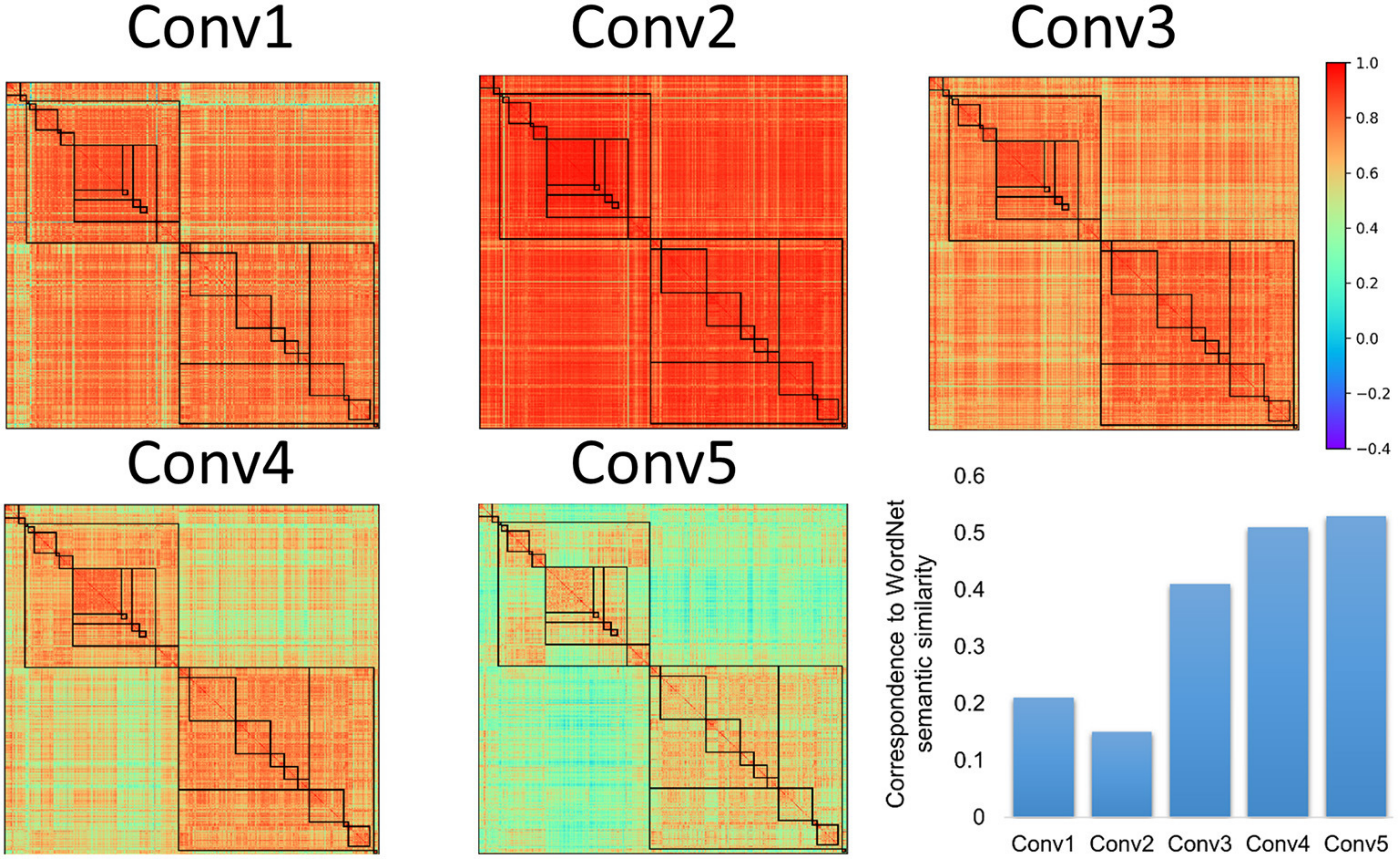
The category representational similarity in different convolutional layers of the AlexNet. Hierarchical relations of objects in the AlexNet gradually emerged as a function of convolutional layers, so was the correspondence between the representational similarity in the AlexNet and WordNet semantic similarity in human. Coarse structure first emerged in lower layers, while the fine-grained structure was prominent only in higher layers.

To test this conjecture, the names of the object categories were put into WordNet derived from human, and their semantic similarity was calculated with the Wu and Palmer's similarity approach (Figure 2B). We found that there was a significant correlation between semantic similarity of WordNet in human and the hierarchical relation among objects in the AlexNet (*r* = 0.56, *p* < 0.001), and correlation also reached significance for both the living thing (*r* = 0.70, *p* < 0.001) and artifact (*r* = 0.41, *p* < 0.001). Similar correspondence to the semantic similarity of WordNet in human was also observed in DCNNs from the VGG family and ResNet family (Figure 3C). In addition, the correspondence of the AlexNet increased as a function of layers (Figure 4), with lower correlations observed in first two layers (layer 1: *r* = 0.21, layer 2: *r* = 0.15) and higher correlations in the third (*r* = 0.41), forth (*r* = 0.51), and fifth (*r* = 0.53) layers. A close inspection on the increases of hierarchy among layers revealed that coarse structure (e.g., the living thing vs. artifact) first emerged in lower layers, and a fine-grained structure was prominent only in higher layers.

**Figure 5:**
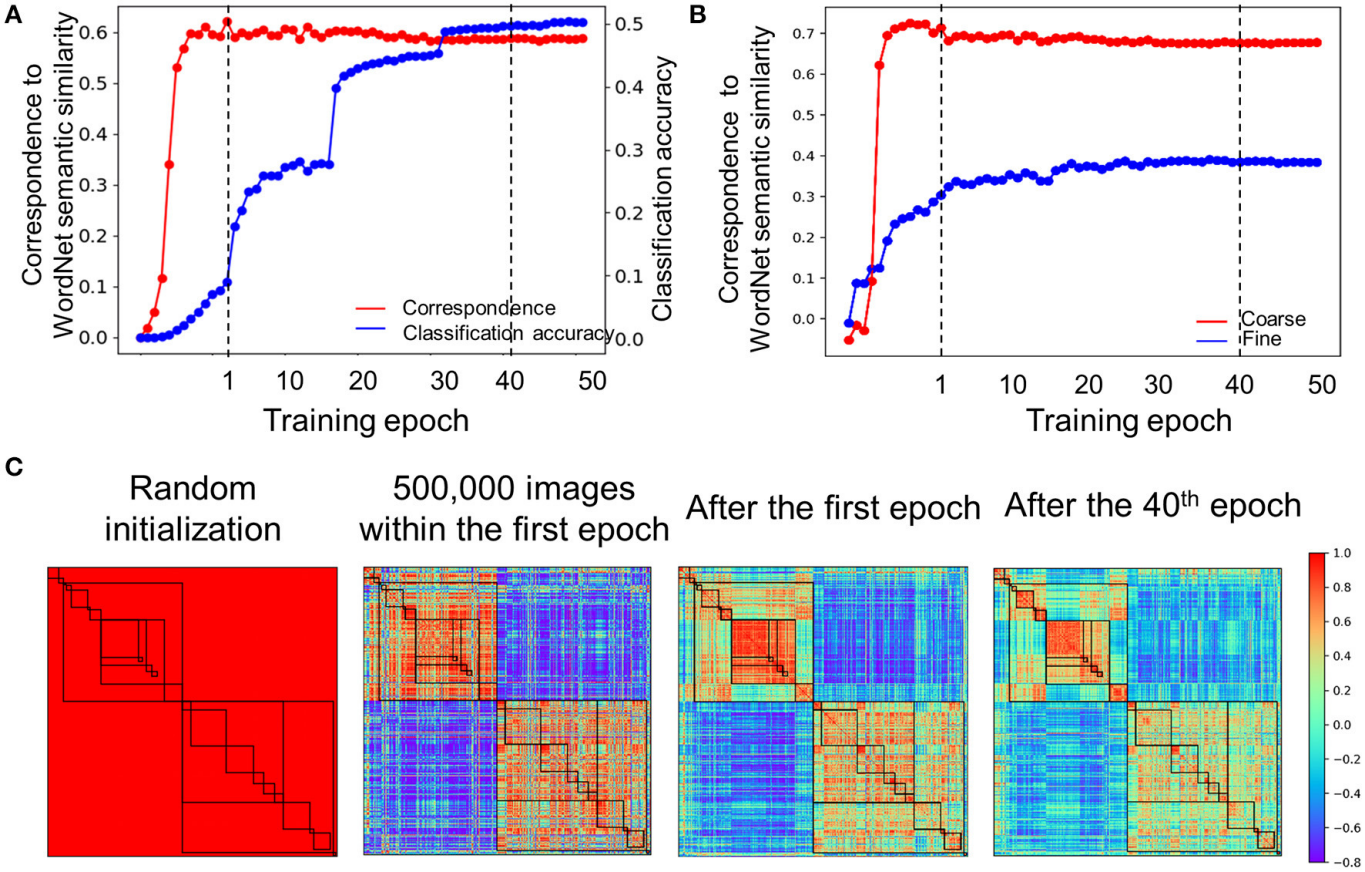
Developmental trajectory of the relatedness. **(A)** The developmental trajectory of the correspondence in the hierarchical relatedness of object categories between the AlexNet and WordNet (red line). The classification accuracy of the AlexNet was shown in blue. The hierarchical structure evolved into maturity far before the establishment of object recognition ability. To illuminate results within the first epoch, correspondence to the WordNet semantic similarity for every 100,000 images was plotted. Dash line indicates epoch 1 and epoch 40, respectively. **(B)** A coarse to fine shift during training progression. The coarse structure based on the 19 superordinate categories reached a plateau within the first epoch (red line), while the fine-grained structure reached a plateau after 40 epochs' training (blue line). Dash line indicates epoch 1 and epoch 40, respectively. **(C)** The category similarities of the AlexNet in different training stages for comparison. From left to right, category similarities of the AlexNet without training, AlexNet trained with 500,000 images within the first epoch, AlexNet trained after the first epoch and AlexNet trained after the 40th epoch. Color bar indicates correlation coefficients.

How did the hierarchical relatedness of object categories emerge from unstructured image dataset in the DCNNs? To address this question, we explored the developmental trajectory of the relatedness when the AlexNet was trained to recognize objects. Two findings were observed. First, correspondence in the hierarchical relatedness of object categories between the AlexNet and the WordNet was established within the first epoch (*r* = 0.60, Figure 5A), whereas the performance for object recognition (top-1 accuracy: 8.9%) was far below that of the fully trained one (top 1 accuracy: 51%). Instead, at least 40 training epochs were needed to attain the matched performance to the fully trained model. The asynchronous development illuminated that the relatedness of object categories in the AlexNet was formed before it was capable of performing the task. Second, within the development of the hierarchical relatedness, there was a progression from a coarse structure to a fine-grained structure. That is, the coarse structure based on the 19 concepts (e.g., bird and device) merged from 1,000 object categories reached a plateau within the first epoch (Figure 5B), with a correlation of 0.65 to the WordNet. In contrast, the fine-grained structure within the 19 concepts (e.g., crane and flamingo in bird) did not approach a plateau until 40 epochs' training, with an averaged correlation of 0.38 to WordNet in humans. Therefore, the hierarchical relatedness of object categories was formed in a coarse-to-fine fashion, with the coarse structure formed before the fine-grained structure (Figure 5C).

**Figure 6:**
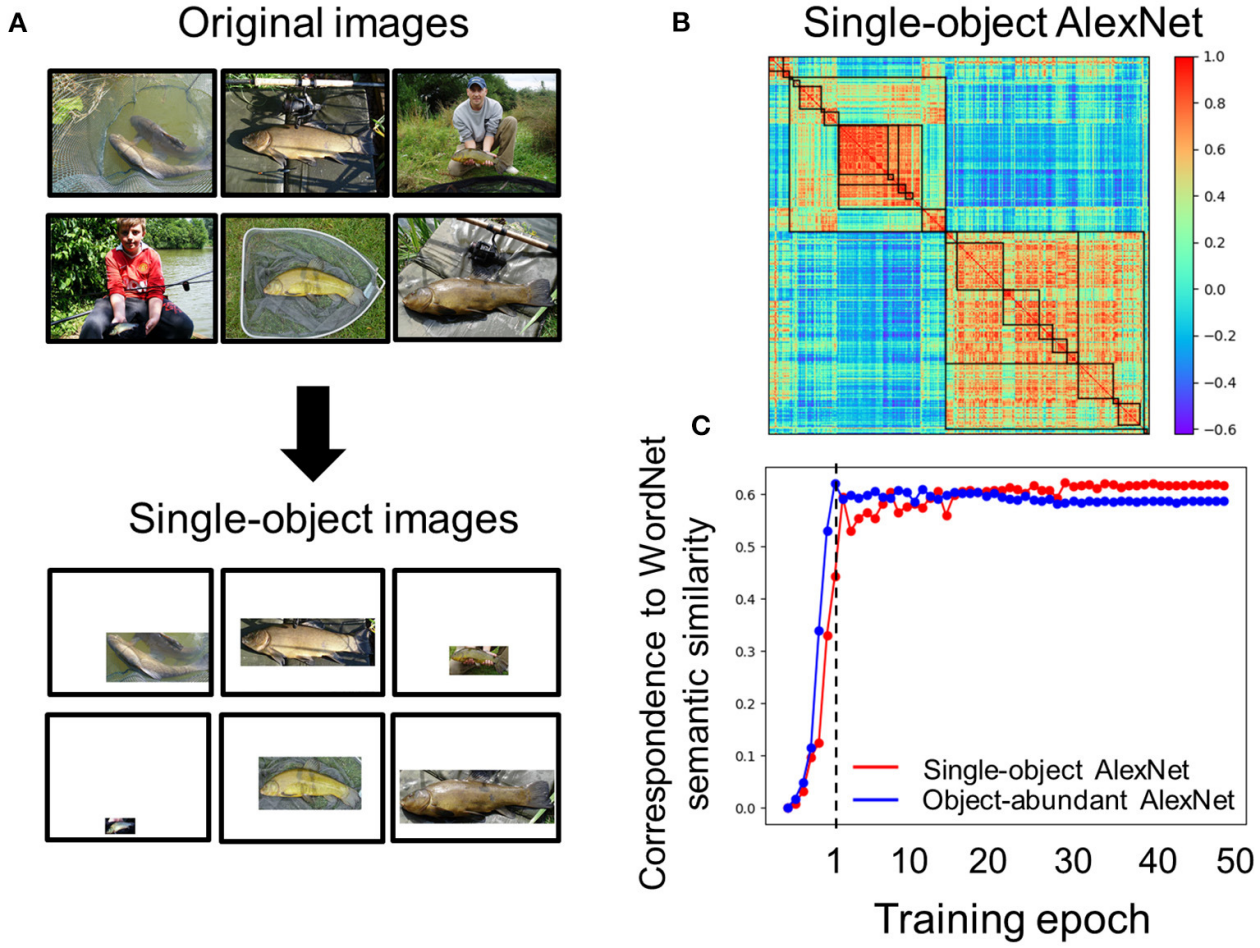
Effect of object co-occurrence on the emergence of semantic relatedness in AlexNet. **(A)** Original images used for training AlexNet contain objects present in the background, which may contribute to the emergence of semantic relatedness in AlexNet. After removing the background, only one object remained. **(B)** Category similarity of the single-object AlexNet, which was trained with images containing only one object. Hierarchical relation is prominent. **(C)** The developmental trajectory of the single-object AlexNet (red) was drawn against that of the original AlexNet (blue). Note that to match the number of images used to train the single-object AlexNet, stages for training the original AlexNet with 600,000 to 1,200,000 images within the first epoch were not plotted.

In natural environments, objects are seldom alone; further, semantically-related ones are often present together. This object co-occurrence may be preserved in images for training DCNNs, and thus contribute to the emergence of semantic relatedness in a DCNN. To rule out this possibility, we trained an AlexNet with images containing a single object without any background (i.e., the single-object AlexNet, see Materials and Methods) (Figure 6A). We found that the hierarchical relation of object categories from the single-object AlexNet was highly correlated with that in the pre-trained AlexNet (*r* = 0.83) (Figure 6B), suggesting that the object co-occurrence was not critical for the emergency of semantic relatedness in DCNNs. In addition, a similar developmental trajectory was also observed (Figure 6C).

**Figure 7:**
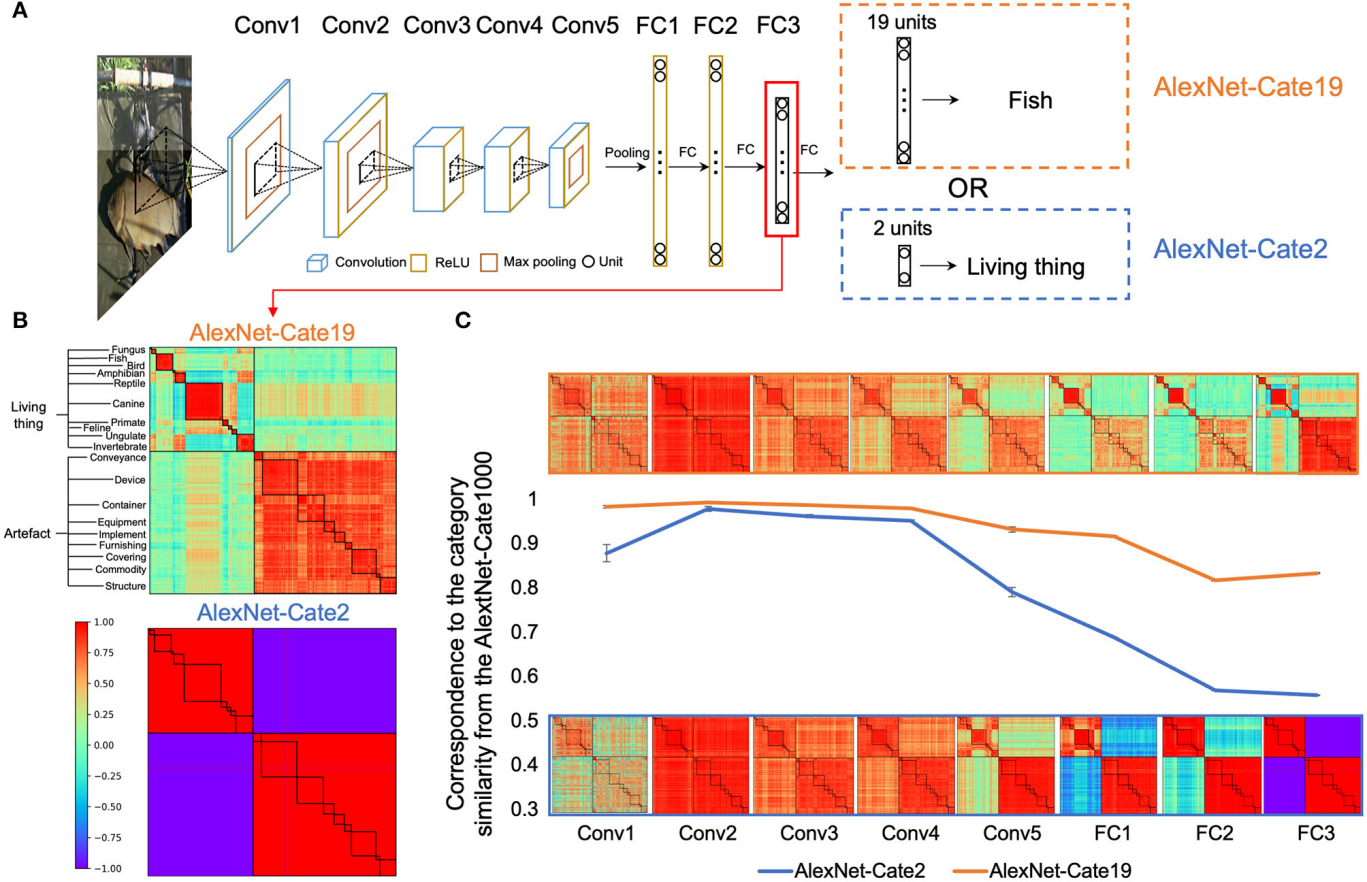
Effect of task demands on semantic relatedness in AlexNet. **(A)** The architectures of AlexNet-Cate19 and AlexNet-Cate2, both of which inherited the same architecture as the original AlexNet, except that one extra FC layer was appended to the FC3 layer. **(B)** Category similarities of AlexNet-Cate19 and AlexNet-Cate2 from the FC3 layer. The hierarchical structures were less prominent in AlexNet-Cate19 as compared to the original AlexNet, and almost absent in AlexNet-Cate2. **(C)** Stimulus-behavior dissociation was formed along the hierarchy of the networks, with the similarity in representation diverging after the fourth convolutional layers. Error bars indicate the standard deviation of the AlexNet-Cate2 and AlexNet-Cate19 after the training was repeated eight times.

Another probable factor that may shape the hierarchy is the task demand, as recent studies suggest behavior-related representations of DCNNs are largely shaped by tasks that DCNNs performed (Song et al., 2020), rather than the physical properties of stimuli (Xu et al., 2020). To test this possibility, we directly compared AlexNet-Cate2 and AlexNet-Cate19 that were designated to classify objects into 2 or 19 superordinate categories, respectively (Figure 7A). The newly added FC layer did not significantly change the internal dynamics of the original AlexNet, as the semantic hierarchy observed in the AlexNet-Cate1000 was almost identical to that of the original AlexNet (*r* > 0.90 for all layers).

We examined the semantic relatedness of the FC3 layer in AlexNet-Cate2 and AlexNet-Cate19, which corresponds to the last layer of the original AlexNet. First, the coarse structure was reserved, as the semantic relatedness emerged in the Alexnet-Cate2 (*r* = 0.65, *p* < 0.001) and AlexNet-Cate19 (*r* = 0.89, *p* < 0.001) was significantly correlated with that in the AlexNet-Cate1000 (Figure 7B). However, the degree of the fineness of the structures differed greatly, as the higher superordinate level of object classification was, the coarser the structure of the relatedness emerged. Importantly, such difference was prominent only at the later layers of the networks (Figure 7C). That is, the relatedness of object categories in the first four layers of AlexNet-Cate2 and AlexNet-Cate19 was similar to that in the AlexNet-Cate1000 (*rs* > 0.89), possibly driven by the physical properties of stimuli. Then, after the fourth layer, their correspondence to AlexNet-Cate1000 decreased gradually, with that of AlexNet-Cate2 decreasing more dramatically. The divergence in correspondence likely reflected the difference in task demands. In short, the stimulus-behavior dissociation that gradually formed along the hierarchy of the networks reflects the joint efforts of stimuli and tasks in shaping the semantic relatedness of object categories.

## Discussions

In this study, we used DCNNs as a model for human cognition to examine whether the semantic relatedness of object categories can automatically emerge without top-down conceptual guidance. First, we found that almost identical hierarchical structures of object categorizes emerged in AlexNet, VGG family, and ResNet family, which were highly similar to the WordNet derived in humans. This result suggests that the relation among object categories can be automatically formed without *a prior* conceptual relationship and independent of implementation hardware. Interestingly, the level of fineness of the semantic relatedness was attributed to the task demands of networks, as the stimulus-behavior dissociation was observed along the hierarchy of network layers. In sum, our study provided the first empirical evidence that even without top-down conceptual guidance, the semantic relatedness of objects can be formed from the joint effort of physical properties of stimuli and task demands of networks.

Unlike studies on humans where perceptual experiences are always intermingled with conceptual guidance, the DCNNs provide a perfect model to demonstrate how perceptual experiences contribute to the construction of relatedness among objects (Peterson et al., 2018). This finding is in line with developmental studies where children prefer to naming objects by referring to their perceptual features, suggesting that the perceptual property of objects play an important role in early accessing lexical knowledge (Imai et al., 1994; Gershkoffstowe and Smith, 2004; Samuelson and Smith, 2005). Further, the emerged semantic relatedness is likely independent of implementation, because the DCNNs and human brain, which differ significantly in hardware, show highly similar hierarchical structures of objects.

The similarity in the semantic structure may result from the similarity in architecture that DCNNs are designed with an architecture similar to the human sensory cortex. Accordingly, similar anatomy may lead to similar functions that give rise to similar structures of the relatedness among objects. For example, the top level of the hierarchy was the living things vs. artifacts, mirroring the axis of the mid-fusiform sulcus that separates the coding of animate objects and artifacts in the brain (Grill-Spector and Weiner, 2014), echoing the proposal that DCNNs are feasible models to understand visual cortex (Yamins et al., 2014; Yamins and DiCarlo, 2016). Indeed, Bao et al. (2020) have found that category-selective regions in the primate inferior temporal cortex are organized to encode the object space constructed by dimensions extracted from DCNNs.

Another and more plausible possibility may be the way by which objects are coded in representational space. In DCNNs, an object is firstly decomposed into multiple features, and mapped to a representational space (Xu et al., 2020). Then, the object is reconstructed from the feature repertoire of the representational space based on the demand of tasks (Xiang et al., 2019; Yang et al., 2019; Song et al., 2020). The representational space allows DCNNs to use the efficient coding scheme (Barlow, 1961; Liu et al., 2020) to reduce the redundancy of the natural stimuli, which is also widely observed in neuroscience studies (Dan et al., 1996; Kastner et al., 2015). Further, features of the representational space are distributedly represented by different units (Liu et al., 2020; Yang and Wang, 2020); therefore, if two objects are perceptually similar because of shared features, they are likely represented by the same set of units. In this way, the relation between two objects is then derived from the connections among units. This intuition is consistent with the hypothesis of parallel distributed processing (McClelland and Rogers, 2003; Saxe et al., 2019), where knowledge arises from the interactions of units through connections. Accordingly, the knowledge stored in the strengths of the connections finally becomes the building blocks of the hierarchical structure of object categories.

Importantly, such hierarchical structure emerged in a coarse-to-fine fashion. That is, at the initial stage of learning, DCNNs may encode global features to identify relations among objects when only a small number of exemplars are available. For example, dogs and cats are the same, but they are not trees based on general appearance. When more exemplars are learned, features in the repertoire are greatly enriched, and thus are capable of providing fine-grained representations for objects to establish the hierarchical structure of relationships among objects. This coarse-to-fine representation is also observed in infants, as infants are able to distinguish animals and vehicles at 7 months old, but fail to differentiate dogs from cats until 11 months old (Mandler and Mcdonough, 1993, 1998; Pauen, 2002).

Interestingly, we also found that the hierarchical structure evolved into maturity before the establishment of object recognition ability. This is not surprising because the enriched and structured feature repertoire is necessary for DCNNs to successfully recognize novel objects never seen before. For example, in a recent study where DCNN's experience on faces is selectively deprived, the DCNN is still capable of accomplishing a variety of face tasks behaviorally and evolving face-specific modules internally (Xu et al., 2020). Therefore, a mature representational space of objects will greatly benefit DCNNs' performance. This mechanism has already widely used in computer science, as transfer learning, for example, utilizes it to harness a pretrained network to work in another domain with a small number of exemplars but still with high accuracy (Olivas et al., 2009).

Besides the physical properties of stimuli, the demand of tasks also played an important role in shaping the representational space of objects especially when it needs to be read out for behavioral performance (Peterson et al., 2018; Turner et al., 2019). When the DCNN was designated to classify objects at superordinate levels rather than at the basic level, the representational space became coarser and the nested structure of the semantic relatedness was less prominent. However, at the earlier layers of the network, the representational space was less likely affected by task demands; rather it was mainly driven by the physical properties of stimuli. As the information flew into later layers, the stimulus-behavior dissociation was observed, as the representational space was mainly shaped by the demand of tasks. Therefore, it is possible that DCNNs extracted images' features based on image statistics into a repertoire to construct a representational space in lower layers, and then only selected features necessary for tasks that the network performed to constructed a new representational space in higher layers. Note that the demand of tasks did not provide any information on the hierachical stucture of objects, and therefore it only shaped the level of fineness of semantic relatedness. Given the similarity in anatomy between DCNNs and primates' systems, future studies are advocated to examine whether primates' visual cortex also follows similar rules to transfer sensation to perception and finally to concepts that lead to behaviors.

In sum, our study demonstrated that perceptual similarity among object categories and the demand of tasks jointly shaped the hierarchical structure among objects. However, there are several limitations to this study. First, this finding did not necessarily rule out the role of conceptual guidance in forming the semantic relatedness, which was clearly illustrated by a moderate correlation between the DCNNs and humans in the hierarchical structure among objects. In addition, the DCNNs used in this study are purely feedforward, and may not be suitable for studies on conceptual guidance. Therefore, other deep neural networks with feedback connections, such as Feedback-CNN or predictive coding network (Lotter et al., 2016; Cao et al., 2018), or networks directly trained with lexical and semantic relations (Bayer and Riccardi, 2016), shall be used to understand how relations between concepts modulate the semantic relatedness of objects without the influence of perceptual experiences. Second, it is counter-intuitive that the semantic relatedness was not derived from object co-occurrence in natural images. That is, it may result from features, rather than co-appearance frequencies, shared by objects. Further studies are needed to examine this hypothesis to unveil the bottom-up mechanism in forming the semantic relatedness of objects.

## Data Availability Statement

The datasets presented in this study can be found in online repositories. The names of the repository/repositories and accession number(s) can be found in the article/supplementary material.

## Author Contributions

TH, ZZ, and JL designed research and wrote the paper. TH performed research and analyzed data. All authors contributed to the article and approved the submitted version.

## Conflict of Interest

The authors declare that the research was conducted in the absence of any commercial or financial relationships that could be construed as a potential conflict of interest.
